# miRNA-148a Enhances the Treatment Response of Patients with Rectal Cancer to Chemoradiation and Promotes Apoptosis by Directly Targeting *c-Met*

**DOI:** 10.3390/biomedicines9101371

**Published:** 2021-10-01

**Authors:** Chun-Ming Huang, Ming-Yii Huang, Yen-Cheng Chen, Po-Jung Chen, Wei-Chih Su, Tsung-Kun Chang, Ching-Chun Li, Ching-Wen Huang, Hsiang-Lin Tsai, Jaw-Yuan Wang

**Affiliations:** 1 Department of Radiation Oncology, Kaohsiung Municipal Ta-Tung Hospital, Kaohsiung Medical University, Kaohsiung 80708, Taiwan; 930321@ms.kmuh.org.tw; 2 Department of Radiation Oncology, Kaohsiung Medical University Hospital, Kaohsiung Medical University, Kaohsiung 80708, Taiwan; miyihu@kmu.edu.tw; 3 Department of Radiation Oncology, Faculty of Medicine, College of Medicine, Kaohsiung Medical University, Kaohsiung 80708, Taiwan; 4 Division of Colorectal Surgery, Department of Surgery, Kaohsiung Medical University Hospital, Kaohsiung Medical University, Kaohsiung 80708, Taiwan; googoogi05@gmail.com (Y.-C.C.); glaudiotennis@gmail.com (P.-J.C.); lake0126@yahoo.com.tw (W.-C.S.); tsungkunchang@gmail.com (T.-K.C.); dobird05@yahoo.com.tw (C.-C.L.); baseball5824@yahoo.com.tw (C.-W.H.); 5 Graduate Institute of Clinical Medicine, College of Medicine, Kaohsiung Medical University, Kaohsiung 80708, Taiwan; 6 Department of Surgery, Faculty of Medicine, College of Medicine, Kaohsiung Medical University, Kaohsiung 80708, Taiwan; 7 Graduate Institute of Medicine, College of Medicine, Kaohsiung Medical University, Kaohsiung 80708, Taiwan; 8 Center for Cancer Research, Kaohsiung Medical University, Kaohsiung 80708, Taiwan; 9 Center for Liquid Biopsy and Cohort Research, Kaohsiung Medical University, Kaohsiung 80708, Taiwan; 10 Ministry of Health and Welfare Pingtung Hospital, Pingtung 90054, Taiwan

**Keywords:** miRNA-148a, rectal cancer, chemoradiotherapy, apoptosis, *c-Met*

## Abstract

Patients with locally advanced rectal cancer (LARC) who achieve a pathological complete response (pCR) to neoadjuvant chemoradiotherapy (NACRT) have an excellent prognosis, but only approximately 30% of patients achieve pCR. Therefore, identifying predictors of pCR is imperative. We employed a microRNA (miRNA) microarray to compare the miRNA profiles of patients with LARC who achieved pCR (pCR group, n = 5) with those who did not (non-pCR group, n = 5). The validation set confirmed that miRNA-148a was overexpressed in the pCR group (n = 11) compared with the non-pCR group (n = 40). Cell proliferation and clonogenic assays revealed that miRNA-148a overexpression radio-sensitized cancer cells and inhibited cellular proliferation, before and after irradiation (*p* < 0.01). Apoptosis assays demonstrated that miRNA-148a enhanced apoptosis before and after irradiation. Reporter assays revealed that *c-Met* was the direct target gene of miRNA-148a. An in vivo study indicated that miRNA-148a enhanced the irradiation-induced suppression of xenograft tumor growth (*p* < 0.01). miRNA-148a may be a biomarker of pCR following NACRT and can promote apoptosis and inhibit proliferation in CRC cells by directly targeting *c-Met* in vitro and enhancing tumor response to irradiation in vivo.

## 1. Introduction

Colorectal cancer (CRC) is the leading cause of cancer mortality worldwide, and approximately 30% of CRC cases are rectal cancer [1]. Neoadjuvant chemoradiotherapy (NACRT) is the standard treatment for patients with locally advanced rectal cancer (LARC) [2,3]. However, the response to NACRT is heterogeneous, ranging from chemoradioresistance to pathological complete response (pCR). Only 15–30% of patients with LARC achieve pCR following NACRT [2,4,5]. Patients with a pCR experience excellent oncological outcomes and may not require adjuvant chemotherapy [6,7]. Therefore, reliable predictive biomarkers of pCR to NACRT must be identified for personalized therapy.

MicroRNAs (miRNAs), non-protein-coding RNAs, regulate the expression of their protein-coding genes by degrading mRNA or repressing translation. miRNAs contribute to several critical biological functions, including carcinogenesis, cell proliferation, and apoptosis [8,9]. They are involved in certain regulatory pathways that mediate cellular radiosensitivity. Liu et al. reported that miRNA-148b promotes radiation-induced apoptosis, thus enhancing radiosensitivity in lymphoma cells [10]. Zhend et al. indicated that radio-resistance in CRC cells was induced by the acquisition of tumor-initiating cell capacity and by the overexpression of miRNA-106b, which directly targets *PTEN* and *p21* [11]. In one study, the overexpression of let-7a deactivated *KRAS* signaling and promoted radiosensitivity in lung cancer cells [12]. miRNA-148a suppresses *VEGF* by downregulating the pERK/HIF-1α/VEGF pathway, which may inhibit angiogenesis in CRC [13]. In summary, the radiosensitivity of cancer cells is regulated by certain miRNAs; they may serve as predictors of tumor response to radiotherapy. However, the clinical implications of these biomarkers have not been elucidated. Herein, we investigated the correlation between miR-148a expression and pCR in patients with LARC following NACRT and determined how miRNA-148a regulates the radiosensitivity of CRC cells.

## 2. Materials and Methods

### 2.1. Patients and Tissue Specimens

**Figure 1:**
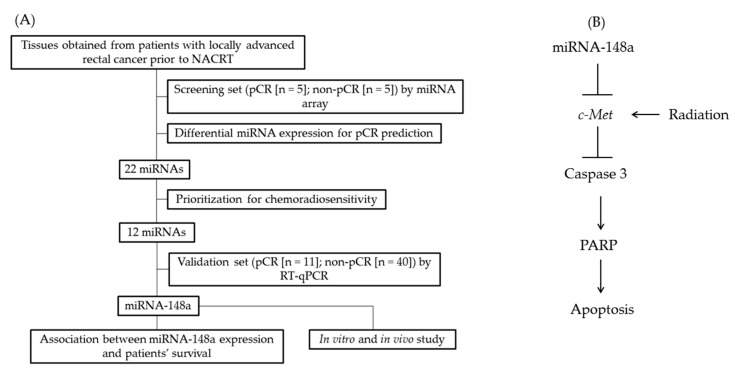
The study design and hypothesis. (**A**) The design of identification of the candidate miRNA. (**B**) The potential regulatory pathway of miRNA-148a.

The study protocol was approved by the Institutional Review Board of Kaohsiung Medical University Hospital (KMUHIRB-02-11-2011). All participants signed an informed consent form. From May 2012 to March 2015, 51 patients with LARC treated with NACRT and radical resection were enrolled, and pretreatment cancer tissues were collected during colonoscopic biopsy and used for miRNA analysis. NACRT consisted of 50 Gy of irradiation concurrently with 5-fluorouracil-based chemotherapy. Radical resection was performed 8–12 weeks after NACRT. A pCR was indicated by the absence of any viable cancer cells in the primary tumor and lymph nodes. Patients were dichotomized according to their pathological response into pCR and non-pCR groups. The design of the identification of the candidate miRNA is shown in Figure 1A, and the potential regulatory pathway of miRNA-148a is illustrated in Figure 1B.

### 2.2. miRNA Microarray

An miRNA microarray (Applied Biosystems, Waltham, MA, USA) containing probes for 667 human miRNAs was used to evaluate and compare the differential expression of miRNAs in the pCR and non-pCR groups. The mammalian U6 small nuclear RNA was used as the internal control for the detected miRNAs. PCR was performed using an Applied Biosystems 7900HT Real-Time PCR System, with default thermal cycling conditions on the ABI 7900 Sequence Detection System version 2.4.

### 2.3. miRNA Expression by RT-qPCR

Total RNA was extracted from harvested cells using MasterPure Complete DNA and RNA Purification Kit Bulk Reagents (cat no. MC85200; Biosearch Technologies, Middleton, WI, USA). For the synthesis of cDNAs specific to miR-148a, a TaqMan MicroRNA Reverse Transcription Kit (cat no. 4366596; Applied Biosystems, Foster City, MA, USA) was used. To determine the gene expression levels, qPCR reactions were performed with a TaqMan Universal Master Mix II kit (cat no. 4440040; Applied Biosystems, Foster City, MA, USA). U6 small nuclear RNA was used as an internal control for miRNA-148a. Relative expression levels were normalized to U6 expression levels to yield a 2^−ΔΔCt^ value.

### 2.4. Putative Target Genes of miRNA-148a

The TargetScan program (www.targetscan.org (accessed on 1 March 2017)) was used to identify the potential target genes of miRNA-148a. Only conserved sequences located in conserved target genes were considered. We used the Gene Ontology (www.geneontology.org (accessed on 18 May 2017)) software to detect the function of the target genes of miRNA-148a.

### 2.5. Cell Culture and Irradiation

Human CRC cell lines, HT29 and HCT116, were purchased from the American Type Culture Collection (Manassas, VA, USA) and the Bioresource Collection and Research Center (Hsinchu, Taiwan), respectively. All cells were cultured in DMEM (Gibco, Grand Island, NY, USA) supplemented with 10% fetal bovine serum (Gibco) and 1% penicillin–streptomycin (Gibco) at 37 °C in a 5% CO_2_-humidified atmosphere. Cells were irradiated with 0, 2, 4, 6, or 8 Gy using an Eleka Axesse medical linear accelerator (Elekta, Crawley, UK). A 1-cm bolus was placed on the top of the culture dish, and cells were irradiated with 6-MV photon beams at 600 MU/min [14].

### 2.6. Cell Transfection

The HT29 and HCT116 cells were seeded in 24-well plates and transfected with 400 ng of miRNA-148a expression vector (pCDH-miRNA-148a) or a negative scrambled pCDH vector by using Lipofectamine 2000 transfection reagent (Thermo Fisher Scientific, Waltham, MA, USA). To select stably transfected cells, we cultured the cells for 4 weeks in selection media supplemented with 10 µg/mL puromycin (Sigma-Aldrich, St. Louis, MO, USA). miRNA expression was measured using a TaqMan miRNA reverse transcription-quantitative polymerase chain reaction (RT-qPCR) assay (Applied Biosystems, Foster City, MA, USA) to confirm stable plasmid transfection. The transfected cell lines were then employed in the subsequent experiments.

### 2.7. Cell Viability Assay

Cell viability was examined using an MTT (3-(4,5-dimethylthiazol-2-yl)- 2,5-diphenyltetrazolium bromide reduction) assay. In brief, stable transfected HT29 and HCT116 cells were seeded at a density of 5 × 10^4^ cells/well in 96-well plates. Subsequently, cells were irradiated with a single dose of 0, 2, 4, 6, or 8 Gy. After 72 h, the culture medium was removed and replaced with 0.5 mg/mL MTT and allowed to stand for 1 h at 37 °C for the formation of purple formazan. The precipitated formazan was dissolved with 100 µL of DMSO, and absorbance was measured at 570 nm with a microplate reader (Thermo Fisher Scientific, Waltham, MA, USA).

### 2.8. Colony Formation Assay

For the clonogenic formation assay, transfected cells were seeded in 6-well plates at a density of 6 × 10^3^ cells/well and exposed to 2–8 Gy of irradiation on day 2. After 10 days of incubation, the colonies were fixed with methanol/acetic acid (3:1) and stained with 0.5% crystal violet in 50/50 methanol/water for 20 min at room temperature. Next, the staining solution was carefully removed from each well and rinsed with water. Finally, the number of cell colonies with a size ≥1 mm was counted using ImageJ software (Java 1.8.0_172).

### 2.9. Cell Cycle and Apoptosis Analysis by Flow Cytometry

After synchronization with serum starvation for 24 h, cells were irradiated at a dose of 4 Gy. Following 4 days of incubation, floating and adherent cells were harvested for cell cycle and apoptosis analysis. For cell cycle analysis, cells were fixed with 75% ethanol at 4 °C overnight. After cells were washed twice with PBS, they were resuspended with PI/Triton X-100 (20 µg/mL PI, 0.1% Triton X-100, and 0.2 mg/mL RNase A) and incubated in the dark for 30 min. To detect apoptosis, we stained the harvested cells with PE-labeled Annexin-V/7-AAD, according to the manufacturer’s protocol (cat no. 559763; BD Biosciences, San Diego, CA, USA). The signals of 1 × 10^5^ stained cells in each sample were detected through flow cytometry (Beckman Coulter, Fullerton, CA, USA).

### 2.10. Western Blotting

*c-Met*, caspase-3, poly (ADP-ribose) polymerase (PARP), and GAPDH were quantified using Western blotting. After 72 h of irradiation, the whole-cell extract was isolated using RIPA buffer (1 mM EDTA [pH 8.0], 100 mM NaCl, 20 mM Tris [pH 8.0], 0.5% Nonidet P-40, and 0.5% Triton X-100). In brief, equal amounts of protein were separated by SDS-PAGE and transferred to polyvinylidene difluoride membranes. Membranes were then incubated with Trident Universal Protein Blocking Reagents (GTX30963; GeneTex, Irvine, CA, USA) for 30 min at room temperature. This was followed by incubation with primary antibodies at 4 °C overnight. Target proteins were probed with the following antibodies: anti-phospho-c-Met, -c-Met, -caspase-3, -PARP (1:1000; Cell Signaling Technology, Danvers, MA, USA). Anti-GAPDH (1:1000; Abcam, Cambridge, MA, USA) was used as a loading control for the whole-cell lysates. Subsequently, the membranes were incubated with a 1:5000 dilution of an HRP-conjugated antibody for 1 h at room temperature. Protein bands were developed using an enhanced chemiluminescence detection reagent, and signals were captured using the ChemiDoc MP Imaging System (Bio-Rad Laboratories, Hercules, CA, USA). ImageJ software was used for protein quantification.

### 2.11. Luciferase Reporter Assay

The predicted miRNA-148a binding site of the *Met* 3′UTR sequence (5′-AGGCCACAAAAACACUGCACUGU-3′) (cat. no. CW306396) or mutant 3′-UTR sequence (5′-AGGCCACAAAAACACACGUGACU-3′) (cat. no. CW306397) was cloned into the pMirTarget vector to construct the wild-type (WT) *c-Met* 3′UTR plasmid or the mutant *c-Met* 3′UTR luciferase plasmid (cat. no. PS100062; OriGene Technologies, Rockville, MD, USA). Cells (1 × 10^5^) were seeded into 24-well plates for 1 day and cultured until the cells reached 70–90% confluence. Subsequently, cells were transfected with WT or mutant-3′UTR luciferase plasmid (0.5 µg) using Lipofectamine 3000 reagent (Invitrogen, Thermo Fisher Scientific, Waltham, MA, USA), according to the manufacturer’s instructions. Luciferase activity was measured 48 h after transfection using a dual-luciferase reporter assay kit. Firefly luciferase activity was normalized to Renilla luciferase activity.

### 2.12. Animal Studies

For tumor implantation, 6-week-old male Balb/c nude mice were obtained from BioLasco Taiwan (Taipei, Taiwan). All animal experiments adhered to the protocols of the Institutional Animal Care and Use Committee of Kaohsiung Medical University (IACUC Approval No: 106083) and were performed according to the Guiding Principles for the Care and Use of Laboratory Animals. The mice were acclimatized for 1 week after arrival under a 12 h:12 h dark/light cycle at 22 ± 1 °C with ad libitum access to food and water. The cells were harvested by trypsinization and washed twice with ice-cold serum-free medium, followed by resuspension in 100 μL of serum-free medium. Into the right flank of each mouse, 2 × 10^6^ cells were subcutaneously injected. On days 12, 15, and 17 after the injection, tumors were irradiated with 15 Gy in three fractions. The tumor size (mm^3^) was measured three times a week and calculated as (length × width^2^)/2. Mice were killed 30 days after the injection of tumor cells.

### 2.13. Statistical Analysis

All values are presented as means ± standard errors of the mean of at least three independent experiments. Student’s *t* tests were conducted to analyze the differences in the expression levels of miRNAs in the pCR and non-pCR groups. Kaplan–Meier survival curves were plotted, and a log-rank test was performed to compare time-to-event distributions. Overall survival (OS) was calculated from the date of diagnosis to death from any cause, and disease-free survival (DFS) was calculated from the date of diagnosis to any recurrence. Receiver operating characteristic (ROC) curve analysis was employed to identify the cutoff value of miRNA-148a to predict pCR. All analyses were performed using JMP software (version 10; SAS Institute, Cary, NC, USA). A *p* of <0.05 was considered significant.

## 3. Results

### 3.1. Demographic Data

The patients’ clinicopathologic characteristics are presented in Table 1. Of the 51 patients with LARC receiving NACRT, the median age was 63 years (range, 28–75 years), and 34 (66.7%) were male. The pCR and non-pCR groups comprised 11 (21.6%) and 40 patients (78.4%), respectively.

### 3.2. Differential miRNA Expression for pCR Prediction

**Figure 2:**
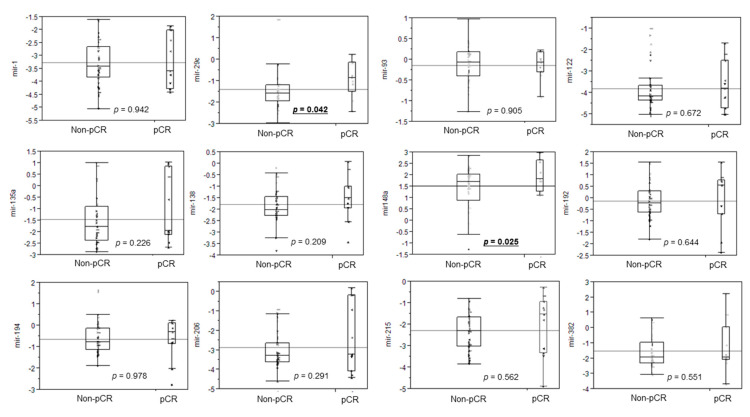
Tissue microRNA (miRNA) levels in 51 patients. To identify clear differences in tissue miRNA levels between the pCR and non-pCR groups, we enrolled 11 patients with a pCR and 40 without a pCR. The dot plots represent 12 miRNA levels quantified by TaqMan real-time polymerase chain reaction (PCR) and normalized to internal controls: U6 level using the 2^−ΔΔCt^ method and stratified by pathological response to neoadjuvant chemoradiotherapy. The horizontal bars represent the medians and 95% confidence intervals. pCR: pathological complete response.

To identify the miRNAs associated with a pCR to NACR, 10 cancer tissues obtained from patients with LARC before NACRT were collected for miRNA microarray analysis. Through this analysis, changes in miRNA expression profiles between the pCR group (n = 5) and the non-pCR group (n = 5) were measured. We observed that 22 miRNAs were differentially expressed in the tissues of patients in the pCR and non-PCR groups. Specifically, 14 were upregulated in the pCR group and 6 were downregulated in the pCR group (Supplementary Table S1). Of the 22 miRNAs, 12 (miRNA-1, miRNA-29c, miRNA-93, miRNA-122, miRNA-135a, miRNA-138, miRNA-148a, miRNA-192, miRNA-194, miRNA-206, miRNA-215, and miRNA-382) were involved in biological pathways for the regulation of cellular chemosensitivity or radiosensitivity. Therefore, we analyzed these 12 miRNAs through TaqMan real-time PCR to identify differences in their expression between the pCR (n = 11) and non-pCR groups (n = 40; Figure 2). miRNA-29c (*p* = 0.042) and miRNA-148a (*p* = 0.025) displayed a more significant overexpression in the pCR group compared with the non-pCR group. Therefore, we selected miRNA-148a as a predictor of pCR and subsequently examined the biological functions of miRNA-148a through in vitro and in vivo studies.

### 3.3. miRNA-148a Overexpression Promoted Radiosensitivity in CRC Cell Lines

**Figure 3:**
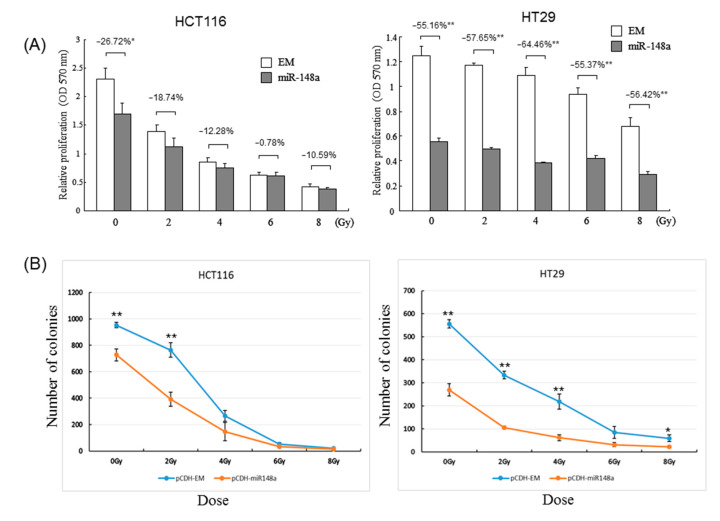
miRNA-148a modulated the proliferation and radiosensitivity of HCT116 and HT29 cells after irradiation. Both cell lines were transfected with a pCDH-miR148a plasmid (miRNA-148a) or a negative scrambled pCDH vector (EM). miR-148a overexpression enhanced the inhibitory effect of cell proliferation (**A**) and colony formation (**B**) after 2-, 4-, 6-, and 8-Gy irradiation (N = 3; * *p* < 0.05; ** *p* < 0.001).

To explore the biological functions of miRNA-148a, we transfected an miRNA-148a mimic into HT29 and HCT116 cells, and miRNA-148a expression was confirmed using RT-qPCR (Supplementary Figure S1). The results of cell viability assays without irradiation indicated that miRNA-148a overexpression significantly inhibited cell growth in both the HT29 and HCT116 cells (both *p* < 0.05, Figure 3A). Next, we exposed the transfected CRC cells to irradiation and conducted cell viability assays. An increased cell death rate following 2-, 4-, 6-, and 8-Gy irradiation was found in the HT29 cells and HCT116 cells (*p* < 0.05). Due to the upregulation of miRNA-148a identified in the pCR group and the inhibition of cell growth following the overexpression and irradiation of the transfected miRNA-148a, we postulated that miRNA-148a could modulate radiosensitivity and, thus, increase the likelihood of a pCR. To further confirm the role of miRNA148a in the enhancement of CRC cells’ radio-sensitivity, we performed clonogenic assays, which revealed that miRNA-148a overexpression significantly reduced colony formation ability following irradiation, compared with that in cells transfected with scrambled miRNAs (*p* < 0.01; Figure 3B).

### 3.4. miRNA-148a Overexpression Led to Cell Cycle Changes in Irradiated CRC Cells

**Figure 4:**
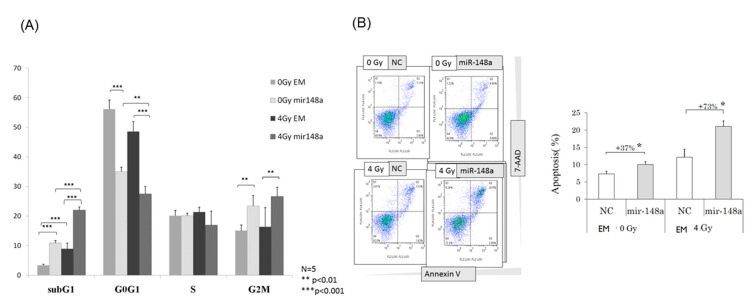
miRNA-148a modulated the cell cycle and promoted apoptosis in HCT116 and HT29 cells after irradiation. After synchronization with serum starvation for 24 h, cells were irradiated with 0 or 4 Gy. Flow cytometry performed after 3 days of incubation indicated that the combination of miR-148a overexpression and irradiation resulted in increased cells in the sub-G1 phase, as well as G2/M arrest (**A**) and an increase in the proportion of apoptotic cells (**B**) (N = 3; * *p* < 0.05; ** *p* < 0.01).

To determine the effects of miRNA-148a on cell cycle alterations, flow cytometry was conducted. A significantly increased G2/M arrest was observed in cells transfected with the miRNA148a mimic after 24 h; furthermore, the increase in the proportion of cells in the G2/M phase was more prominent in cells overexpressing miRNA148a and subjected to 4-Gy radiation (*p* < 0.01; Figure 4A). Similarly, miRNA148a overexpression significantly increased the proportion of cells in the sub-G1 phase, regardless of whether radiation was performed (*p* < 0.001). By contrast, miRNA148a overexpression corresponded to a substantial reduction in the proportion of cells in the G1 phase, whereas miRNA148a overexpression exerted no influence on S-phase alterations.

### 3.5. miRNA-148a Overexpression Enhanced Radiation-Induced Apoptosis in CRC Cells

**Figure 5:**
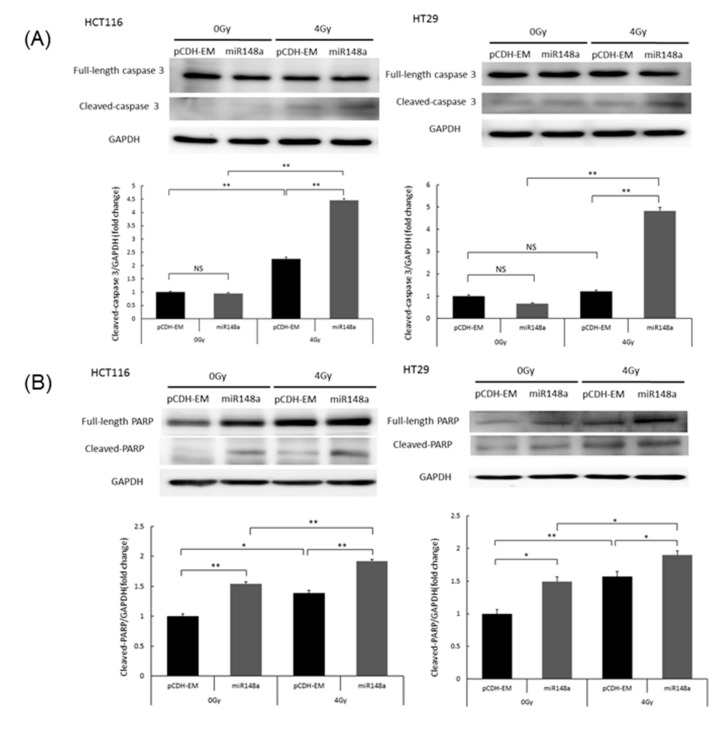
miRNA-148a promoted apoptosis in irradiated HCT116 and HT29 cells. Following irradiation with 0 or 4 Gy, the cells were harvested, and the total protein was extracted for Western blotting. (**A**) miR-148a enhanced the irradiation-induced upregulation of cleaved caspase-3 (N = 3; * *p* < 0.05; ** *p* < 0.01). (**B**) miR-148a enhanced the irradiation-induced upregulation of cleaved poly (ADP-ribose) polymerase (N = 3; * *p* < 0.05; ** *p* < 0.01).

To explore the effects of miRNA-148a on apoptosis, HT29 cells with miRNA148a overexpression were exposed to 4 Gy of radiation and subjected to Annexin-V/7-AAD staining for of the evaluation of apoptosis. miRNA-148a overexpression had a 37% higher increase in apoptotic cells compared with the negative control (NC) groups (*p* < 0.05). The percentage of apoptotic cells in the miRNA148a overexpression group after radiation was significantly higher than that in the control group (*p* < 0.05; Figure 4B). The results indicate the synergistic effects of miRNA148a overexpression with irradiation on apoptosis in CRC cells. To further assess this synergistic effect, we examined apoptosis-related protein markers. Caspase-3 is involved in both extrinsic and intrinsic pathways and, therefore, is the most essential executioner caspase [15]. As presented in Figure 5A, overexpressed miRNA-148a did not activate caspase-3 cleavage, but the combination of miRNA-148a overexpression and irradiation significantly increased caspase-3 cleavage; this implies their synergistic action (*p* < 0.01). Cleaved PARP-1 is a well-established apoptotic marker and indicates an apoptotic-specific event [16]. Figure 5B indicates that miRNA-148a overexpression increased the proportion of cleaved PARP compared with that in the NC groups, and the combination of miRNA-148a and irradiation resulted in the highest levels of cleaved PARP among the groups (*p* < 0.01). Taken together, these findings indicate that miRNA-148a can enhance radiation-induced apoptosis.

### 3.6. miRNA-148a Overexpression Promoted Apoptosis in CRC Cells by Directly Targeting c-Met

**Figure 6:**
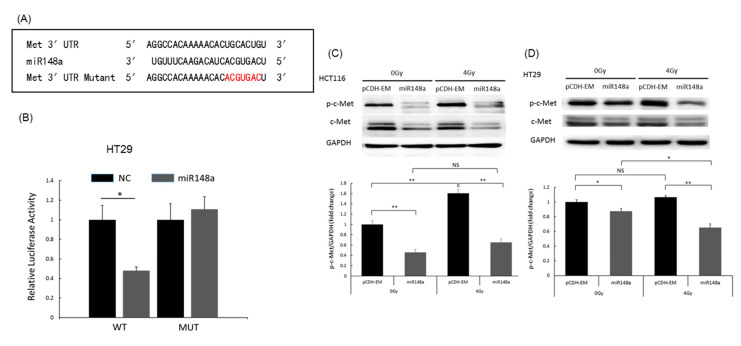
*c-Met* is a target gene of miRNA-148a and is mediated in CRC cells. (**A**) The complementary sequences of miRNA148a and *c-Met*, as well as the mutant sequences of the *c-Met* binding site. (**B**) Wild-type *c-Met* binds to miRNA-148a, whereas the MUT does not. (**C**) In HCT116 cells, *c-Met* expression changed with miRNA-148a expression and irradiation. (**D**) In HT29 cells, *c-Met* expression changed with miRNA-148a expression and irradiation. (N = 3; * *p* < 0.05; ** *p* < 0.01). NC: negative control; MUT: mutant.

The potential target gene of miRNA-148a was explored using the TargetScan program. The analysis indicated the presence of a conserved binding site of miRNA-148a in the 3′UTR of *c*-*Met* (Figure 6A). The luciferase reporter assay demonstrated that the relative luciferase activity of the reporter vector containing WT *c*-*Met*-3′UTR was significantly suppressed by miRNA-148a. By contrast, miRNA-148a did not influence the relative luciferase activity of the mutated reporter (*p* < 0.001; Figure 6B). Moreover, miRNA-148a overexpression significantly inhibited *c-Met* protein expression (*p* < 0.01; Figure 5). In HCT116 cells with pCDH-NC, irradiation induced upregulation of phosphorylated *c-Met*, which was suppressed by miRNA-148a (Figure 6C). However, in HT29 cells transfected with pCDH-NC, irradiation did not upregulate phosphorylated *c-Met* (Figure 6D).

### 3.7. miRNA-148a Overexpression Enhanced Tumor Response to Radiation in Nude Mice

**Figure 7:**
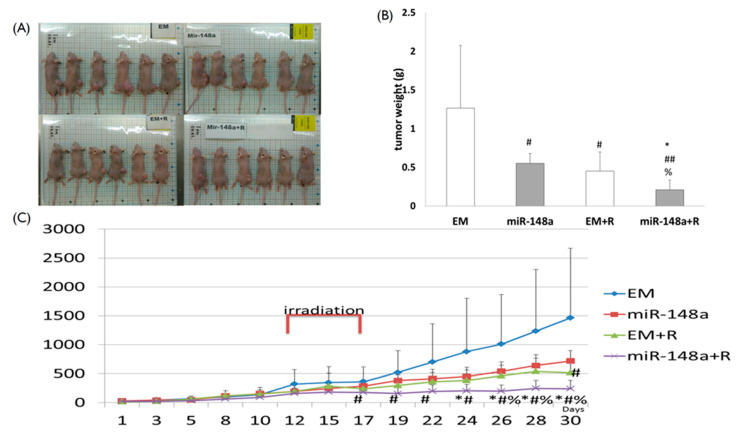
miR-148a enhanced CRC xenograft response to irradiation. HT29 cells stably transfected with miR-148a or a negative scrambled pCDH vector (EM) were injected subcutaneously into the right flank of the mice. (**A**) When tumors reached approximately 100 mm^3^ in volume, mice were randomly assigned to four groups of six and each tumor lump was treated with 15 Gy in 3 fractions. (**B**) Tumor weights (gram). (**C**) Tumor growth volume (mm^3^) was measured with a Vernier caliper on the indicated days after inoculation. (N = 6; # *p* < 0.05, ## *p* < 0.01 compared with EM; * *p* < 0.05 compared with EM + R; % *p* < 0.05 compared with miR148a).

To determine whether miRNA-148a could radio-sensitize CRC cells in vivo, we subcutaneously injected HT29 cells transfected with miRNA-148a or scrambled miRNAs into the right flanks of the mice. When tumors reached a volume of 100 mm^3^, mice were randomly assigned to four groups of six: (1) mice injected with cells containing pCDH-NC; (2) mice injected with cells containing miRNA-148a; (3) mice injected with cells containing pCDH-NC and receiving tumor irradiation; and (4) mice injected with cells containing miRNA-148a and receiving tumor irradiation (Figure 7A). Tumors were irradiated with 15 Gy in three fractions on days 12, 15, and 17 after inoculation. Among the four groups, the volume of cancer lumps with miRNA-148a overexpression in mice receiving irradiation was the smallest (*p* < 0.01; Figure 7B,C). The in vivo results demonstrated that miRNA-148a enhanced tumor response to irradiation, thus further supporting the postulation that miRNA-148a overexpression might enhance radiosensitivity in CRC cells.

### 3.8. miRNA-148a Overexpression Is Associated with a Favorable Prognosis in Patients with LARC Following NACRT

**Figure 8:**
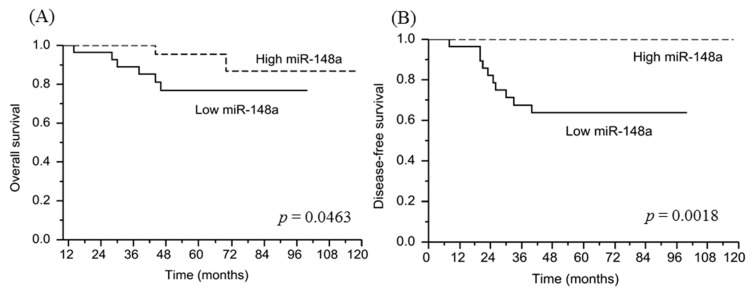
Survival curves of the 51 patients. (**A**) Overall survival in patients with low miRNA-148a expression was significantly poorer than that in those with miRNA-148a overexpression (*p* = 0.0463). (**B**) Disease-free survival in patients with low miRNA-148a expression was significantly shorter than that in patients with miRNA-148a overexpression (*p* = 0.0018).

To examine the clinical significance of miRNA-148a expression in predicting a pCR in patients with LARC, we performed ROC analysis to identify the miRNA-148a cutoff value. The area under the curve was 0.65 (*p* < 0.001) for pCR, and an miRNA-148a expression level of 1.8 corresponded to an optimal sensitivity of 55% and specificity of 58%. We then determined whether the cutoff value was associated with patient outcomes, observing that survival was significantly reduced in patients with low miRNA-148a expression. The 5-year OS rates in the high miRNA148a expression and low miRNA148a expression groups were 95.5% and 76.7%, respectively (*p* = 0.0463; Figure 8A), and the 5-year DFS rates were 100% and 63.8%, respectively (*p* = 0.0018; Figure 8B).

## 4. Discussion

Studies have indicated that NACRT improves prognosis in patients with LARC; however, treatment response varies from total regression to total resistance [2,3]. As patients with a pCR following NACRT have excellent outcomes, a watch-and-wait strategy is recommended in such instances, to prevent surgical sequelae and complications [6,17]. Regarding precision medicine in LARC, identifying molecular biomarkers for accurate pCR prediction is of utmost clinical importance [18,19]. Herein, we demonstrated that miRNA-148a overexpression in cancer tissues before NACRT was associated with a pCR and higher survival rates in patients with LARC following NACRT. Furthermore, miRNA-148a overexpression sensitized CRC cells to irradiation in vitro and in vivo by promoting cancer cell apoptosis through the direct targeting of *c-Met*. Taken together, the results indicate that miRNA-148a can serve as a potential predictive biomarker to guide the watch-and-wait strategy suggested for patients with LARC following NACRT.

miRNAs play an integral role in cancer development and progression and can be classified as oncomiRNAs or tumor suppressor miRNAs on the basis of their biological functions [8]. Moreover, they are potential biomarkers of prognosis or treatment response in many types of cancer, including CRC. Lopes-Ramos et al. analyzed miRNA profiles in 43 rectal tumors prior to NACRT, reporting that miRNA-21-5p was associated with complete tumor regression [20]. Kral et al. observed that the expression of the miR-17/92 cluster was associated with posttreatment regression in patients with rectal cancer [21]. In this study, correlations between miRNA profiles of rectal cancer tissues and their treatment responses were examined, and miRNA-148a expression was found to be related to pCR.

Owing to the overexpression of miRNA-148a in the pCR group compared with that in the non-pCR group, this was regarded as associated with pCR. miRNA-148a, which is located at chromosome 7p15, functions as a tumor suppressor miRNA and is involved in various cancer-related processes, including cell proliferation, invasion, migration, and apoptosis [9]. Studies have noted miRNA-148a downregulation in gastrointestinal, breast, urogenital, and non-small-cell lung cancer. Notably, this downregulation has been associated with reduced survival in CRC and urogenital cancer [22,23]. In line with previous studies, we observed that miRNA-148a overexpression was associated with a pCR following NACRT and improved survival in patients with LARC. In addition, our study demonstrated that overexpressed miRNA-148a in CRC cells inhibited cell growth and induced apoptosis in vitro, as well as inhibiting tumor growth in vivo, even in the absence of radiation. This supports the premise that miRNA-148a acts as a tumor suppressor miRNA.

To investigate whether miRNA-148a functioned consistently in cells bearing distinct gene mutations, we examined the biological functions of miRNA-148a by using two CRC cell lines with distinct mutational statuses [24]. HT29 cells are more radioresistant, whereas HCT116 cells are more radiosensitive [25,26]. Herein, the radio-sensitization of miRNA-148a was more prominent in the HT29 cells than in the HCT116 cells. Moreover, radiation induced the upregulation of *c-Met* in the HCT116 cells, but not in the HT29 cells. This may be attributable to the differences in their mutational statuses. Bacco et al. demonstrated that the irradiation-induced expression of *c-Met* was related to the activation of ATM and NF-kB [27]. Lin et al. analyzed 167 CRC specimens, detecting an association between *NF-κB* activation and *KRAS* mutation [28]. *KRAS* is a mutation in HCT116 cells but is WT in HT29 cells [24]; therefore, we speculated that irradiation-induced *c-Met* upregulation was prominent in the HCT116 cells and not the HT29 cells because *NF-κB* activation might be related to *KRAS* mutation.

The role of miRNA-148a in the regulation of radiosensitivity has rarely been investigated. Wang et al. found that SNHG12, a class of long noncoding RNAs, mediated the radiosensitivity of cervical cancer cells through the miRNA-148a/CDK1 pathway [29]. Lopez-Bertoni et al. observed that the codelivery of miRNA-148a and miRNA-296-5p inhibited the stemness of glioblastoma cells in vitro and enhanced tumor response to irradiation in vivo [30]. In this study, we observed that upregulation of miRNA-148a sensitized CRC cells to irradiation in vitro and in vivo, supporting our postulation that miRNA-148a was associated with pCR (given that it functioned as a radiosensitizer in CRC cells).

Aberrantly regulated *c-Met* is common in gastrointestinal cancer and is considered to be associated with tumor progression and poor survival. *c-Met* is a receptor tyrosine kinase that binds to hepatocyte growth factor and triggers various cancer-associated processes, including proliferation, angiogenesis, invasion, and epithelial–mesenchymal transition [31]. *c-Met* overexpression in patients with CRC has been associated with reduced survival and increased risk of distant metastasis [32]. The present findings indicate that *c-Met* is an miRNA-148a target gene in CRC cells. Furthermore, the combination of miRNA-148a overexpression and irradiation significantly inhibited the expression of *c-Met*, which subsequently promoted apoptosis. *c-Met* is associated with radio-resistance. In one study, its inhibition led to radio-sensitization in various cancers, including CRC [33]. Lal et al. reported that the inhibition of the *c-Met* pathway sensitized glioblastoma to irradiation, both in vitro and in vivo [34]. Cuneo et al. demonstrated that crizotinib, a *c-Met* inhibitor, radio-sensitized *KRAS*-mutant CRC cell lines, suggesting that crizotinib can be prescribed to patients with CRC requiring radiotherapy [35]. Bacco et al. demonstrated that *c-Met* overexpression increased invasiveness and inhibited apoptosis in breast cancer cells and that *c-Met* inhibitors reversed these effects, indicating radio-sensitization in cancer cells by inhibition of *c-Met* [27]. Kawamura et al. analyzed 52 patients with LARC following NACRT and surgery, reporting that *c-Met* overexpression in surgical specimens resulted in poor relapse-free survival [36]. Consistently, the present data indicate that the downregulation of *c-Met* by miRNA-148a enhanced radiosensitivity in tumor cells. Taken together, these results suggest that miRNA-148a, which downregulates *c-Met* expression, is a potential therapeutic agent and radiosensitizer in patients with LARC receiving NACRT. Future studies should confirm the role of miRNA-148a in this regard and address the relevant clinical implications.

Some limitations of this study need to be addressed. First, the number of patients was relatively small. A larger cohort is essential to validate the predictive value of miRNA-148a in LARC. Second, the detailed *c-Met* signaling pathway of mediating radiosensitivity was not completely explored in this study. Activation of *c-Met* induces various cellular signaling pathways and consequent biologic functions. A better understanding of the *c-Met* signaling pathway would help the development of new therapeutic agents. Therefore, the detailed mechanisms of *c-Met*-mediated cellular response to irradiation warrant further studies. Despite these limitations, we consider that miRNA-148a is a potential predictive biomarker and may play an important role in personalized therapy for patients with LARC.

## 5. Conclusions

In this study, we demonstrated that miRNA-148a is a potential biomarker for predicting pCR following NACRT and that it was associated with favorable oncological outcomes in patients with LARC. miRNA-148a overexpression promoted apoptosis and inhibited proliferation in CRC cells by directly targeting *c-Met* in vitro and enhancing tumor response to irradiation in vivo. Further studies on the clinical implications and regulatory mechanism of miRNA-148a are warranted to determine its role in LARC treatment.

## Figures and Tables

**Table 1:** Clinicopathologic Characteristics of the 51 Rectal Cancer Patients Receiving Chemoradiotherapy.

Variables	Numbers (%)
Age, median (range, years)	63 (28–75)
Sex (male/female)	34 (66.7)/17 (33.3)
Histology (WD/MD/PD)	8 (15.7)/40 (78.4)/3 (5.9)
Tumor stage (T2/T3/T4)	8 (15.7)/32 (62.7)/11 (21.6)
Nodal stage (N1/2)	12 (23.5)/16 (31.4)/23 (45.1)
Treatment response (pCR/non-pCR)	11 (21.6)/40 (78.4)

Abbreviations: MD, moderate differentiation; pCR, pathological complete response; PD, poor differentiation; WD, well differentiated.

## Data Availability

The data used to support the findings of this study are included within the article and the data sources are available from the corresponding author upon request.

## Supplementary Materials

The following are available online at https://www.mdpi.com/article/10.3390/biomedicines9101371/s1, Table S1: The microRNA microarray data, Figure S1: miRNA-148a level after pCDH-miRNA-148a vector transfected into HCT116 and HT29.
