# Supplementary material for: miRNA-148a Enhances the Treatment Response of Patients with Rectal Cancer to Chemoradiation and Promotes Apoptosis by Directly Targeting c-Met
Source: Biomedicines. 2021 Oct 1;9(10):1371. doi: 10.3390/biomedicines9101371 (PMC8533359; doi:10.3390/biomedicines9101371)
Supplement: Supplementary file 1 [file biomedicines-09-01371-s001.zip › biomedicines-1379159-supplementary.pdf]

Supplementary Table S1. The microRNA microarray data. We found that 22 miRNAs differentially expressed between pCR tissues and non-pCR tissues (All P < 0.05); 14 miRNAs were upregulated in the pCR group and 6 miRNAs were downregulated in the pCR group.*downregulated miRNAs.

Candidates	Fold change	P value	Candidates	Fold change	P value
hsa-miR-1	3.88	0.031	hsa-miR-122	1.93	0.038
hsa-miR-135a	3.52	0.022	hsa-miR-382	2.66	0.014
hsa-miR-206	6.78	0.007	hsa-miR-3673	3.52	0.008
hsa-miR-138	5.34	0.004	hsa-miR-4710	4.98	0.004
hsa-miR-29c	6.45	0.005	*hsa-miR-192	2.15	0.029
hsa-miR-205	2.18	0.033	*hsa-miR-194	2.58	0.012
hsa-miR-323a	3.79	0.005	*hsa-miR-215	4.98	0.002
hsa-miR-148a	7.17	0.003	*hsa-miR-601	2.06	0.026
hsa-miR-93	2.98	0.016	*hsa-miR-3195	2.31	0.028
hsa-miR-645	3.11	0.029	*hsa-miR-3187	1.56	0.031

Supplementary Figure S1

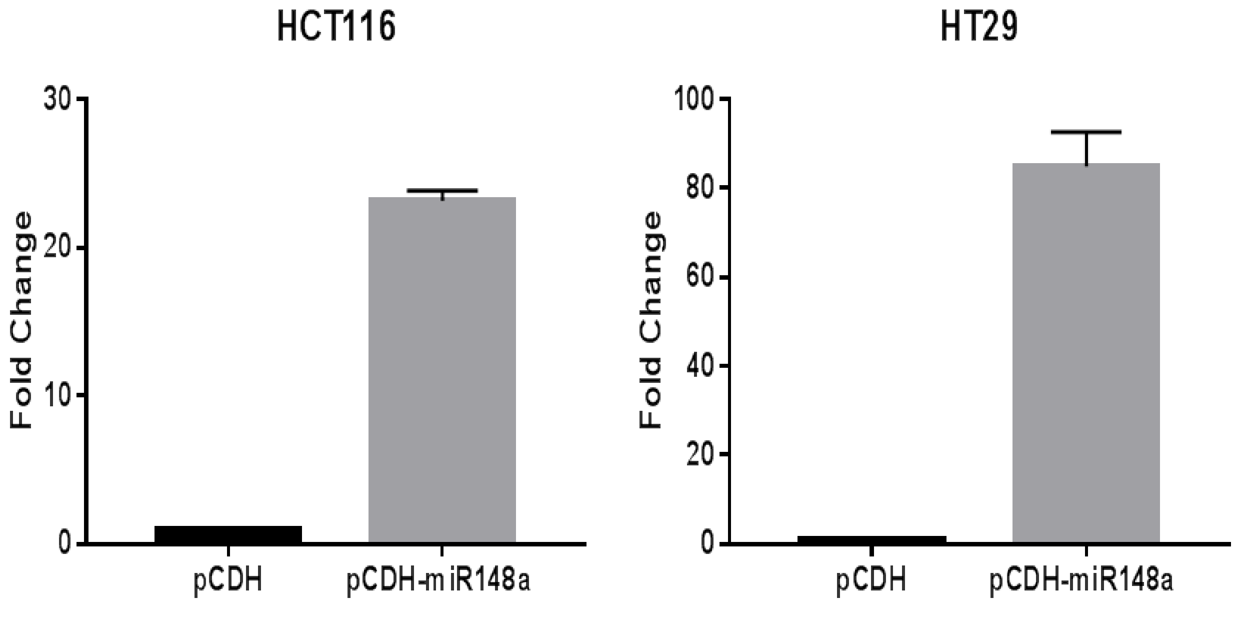

Supplementary Figure S1. Cell lines transfection. Either pCDH-miRNA-148a vector or a negative scrambled pCDH vector was transfected into HT29 and HCT116 cells, and miRNA-148a expression was confirmed using RT-qPCR
